# Higher incidence of persistent chronic infection of *Chlamydia pneumoniae *among coronary artery disease patients in India is a cause of concern

**DOI:** 10.1186/1471-2334-7-48

**Published:** 2007-05-30

**Authors:** Hem C Jha, Harsh Vardhan, Rishein Gupta, Rakesh Varma, Jagdish Prasad, Aruna Mittal

**Affiliations:** 1 Division of Tissue Culture/Microbiology, Institute of Pathology (ICMR), Safdarjung Hospital Campus, New Delhi, India; 2 Department of Cardiology, Safdarjung Hospital Campus, New Delhi, India; 3 Department of Cardiovascular Thoracic & Vascular Surgery, Safdarjung Hospital, New Delhi, India

## Abstract

**Background:**

There is growing evidence that *Chlamydia pneumoniae *may be involved in the pathogenesis of atherosclerosis, as several studies have demonstrated the presence of the organism in atherosclerotic lesions. *C. pneumoniae *infections, which are especially persistent infections, have been difficult to diagnose either by serological methods or isolation of the organism from the tissue. Nucleic Acid Amplification tests (NAATs) has emerged as an important method for detecting *C. pneumoniae*. Inspite of high prevalence of *C. pneumoniae *specific antibodies in coronary heart disease patients, direct detection of *C. pneumoniae *in circulating blood of coronary artery disease (CAD) patients by sensitive nucleic acid amplification tests nested PCR (nPCR), multiplex PCR (mPCR) has not been carried out is required. Further correlation of the presence of *C. pneumoniae *in blood of CAD patients with *C. pneumoniae *specific IgA and IgG antibodies, which may indicative of the status of infection with the progression of atherosclerosis. This will help in order to prepare strategies for the antibiotic intervention to avoid the progression towards CAD.

**Methods:**

Venous blood was obtained from 91 CAD patients and 46 healthy controls. Nucleic acid amplification tests *viz*. nested -, semi-nested – and multiplex PCR were used for detection of *C. pneumoniae*. ELISA carried out prevalence of *C. pneumoniae *specific IgG and IgA antibodies.

**Results:**

29.67% (27/91) patients were positive for *C. pneumoniae *using nested PCR. The sensitivity and specificity of semi-nested and multiplex PCR were 37.03%, 96.96% and 22.22%, 100% with respect to nested PCR. Positive nPCR patients were compared with presence of *C. pneumoniae *specific IgA, IgA+IgG and IgG antibodies. Among 27 (29.67%) nPCR *C. pneumoniae *positive CAD patients, 11(12%) were IgA positive, 13(14.2%) were IgA+IgG positive and only1 (1.1%) was IgG positive. A significant presence of *C. pneumoniae *was detected in heavy smokers, non-alcoholics and with family histories of diabetes and blood pressure group of CAD patients by nPCR.

**Conclusion:**

The results indicate synergistic association of *C. pneumoniae *infection and development of CAD with other risk factors. We also detected increased positivity for *C. pneumoniae *IgA than IgG in nPCR positive CAD patients. Positive nPCR findings in conjunction with persisting high *C. pneumoniae *specific antibody strongly suggest an ongoing infection.

## Background

Coronary artery disease (CAD) is a major cause of morbidity and mortality in humans and is predicted to be the leading cause of death in the world [1]. Acquired metabolic abnormalities like hypercholesterolemia, diabetes mellitus are major risk factors associated with CAD, besides inheritance. These factors on compounding with pathogens like *C. pneumoniae, Helicobacter pylori *and Cytomegalovirus intensifies the magnitude of risk impending towards CAD [2,3]. Several reports have suggested a role of chronic *C. pneumoniae *infection in pathogenesis of CAD and other atherosclerotic syndromes [4,5]. *C. pneumoniae *has been established as an important pathogen that causes infections of upper and lower respiratory tract [6-8]. *C. pneumoniae *has a large amount of factual data that suggests that the organism plays a contributory role in atherosclerosis [4]. These data are based on serology, animal model studies, direct detection of the organism in atherosclerotic lesion, and preliminary clinical trials showing improved outcome among patients treated with antibiotics [9-11]. Changes in habits (Drinking, smoking, junk foods) and habituation (urbanization, migration) also conferred their role in extending predisposition towards CAD [12].

It is reported that Indians have the highest risk of CAD and the prevalence of CAD in India has recently been estimated to be 11% [13]. In a study from India, species-specific *C. pneumoniae *IgG antibodies were detected in 73.7% of CHD patients by immunocomb assay [14]. The high prevalence of *C. pneumoniae *specific serum immunoglobulins is suggestive of an alarming condition of infection in the country and calls for an immediate requirement to ascertain the status of infection in CAD patients.

Detection of *C*. *pneumoniae *in atheromatous plaques was shown by PCR, transmission electron microscopy (TEM), in situ hybridization (ISH) and Immunohistochemistry (IHC) [15], however, the detection of *C*. *pneumoniae *in circulating blood by nucleic acid amplification tests is technically more feasible approach since discrepancies have been reported for detection in vessel walls of specimen [16]. In both the mouse and rabbit models of *C. pneumoniae*-induced atherosclerosis, *C. pneumoniae *has been detected in the blood prior to its appearance in atheromatous lesions of major blood vessels, including the aorta and coronary arteries [17].

Therefore in the present study detection of *C. pneumoniae *was carried out in venous blood of CAD patients using various nucleic acid amplification tests (NAATs) viz; nested PCR (nPCR), semi-nested PCR (snPCR) and multiplex PCR (mPCR). Our first aim was thus to find a reliable NAAT test that would give help in precise detection of *C. pnuemoniae *in blood. Our second aim was to quantify the minimum amount of DNA required for detection of *C. pneumoniae*. As increased IgA antibody levels often reflects an active or persistent infection, whereas IgG levels reflect only a previous or past infection with *C pneumoniae *[18], our third aim was to correlate the presence of *C. pneumoniae *in blood of CAD patients with *C. pneumoniae *specific IgA and IgG antibodies. The early detection of *C. pneumoniae *in venous blood by a sensitive, reliable and economic assay will help to improve patient management in India.

## Methods

### Enrollment of patients

During the period from March 2005 – 2006, 91 patients (72 males and 19 females) attending Cardiology out patient department of Safdarjung hospital, suspected of coronary artery disease and subsequently admitted for angioplasty were enrolled for the study. In addition, 46 age matched healthy controls were also included in the study. The study received clearance from Ethical Committee of Safdarjung hospital. Prior written consent was also obtained from all the patients as well as controls. A detailed questionnaire including reasons for referral to cardiology department, age, sex, smoking habit, alcohol consumption and history of heart disease, blood pressure, or diabetes was maintained for all the patients included in the study.

### Collection of samples

Venous blood (5 ml) was collected in heparinized tubes (Heparin-Hi Media, Mumbai, India) and 2 ml in non- heparinized tubes from CAD patients and controls. Serum was separated and kept at -20°C until analyzed for the detection of *C. pneumoniae *specific IgA and IgG.

### DNA extraction

5 ml of blood sample was incubated with equal amount of sample buffer containing 30 mM Tris-HCl [pH 8.0], 30 mM EDTA, 150 mM NaCl, 0.5% SDS, 0.5% Triton X-100, 5% Tween-20, and proteinase K at 55°C for 3 hrs. Subsequently samples were extracted with phenol: chloroform: isoamyl alcohol (25: 24: 1), followed by precipitation of DNA in ice-cold absolute ethanol and were resuspended in autoclaved distilled water. Quantitation of DNA was performed on a spectrophotometer (Thermo Spectronic, Rochester, New York, USA) and was checked for its integrity on 0.5 % agarose gel before primer specific amplification.

### Primer selection

The major outer membrane protein (*Omp1*) and 16S rRNA genes were the targets for amplification of *C. pneumoniae *specific nucleic acid for snPCR and nPCR. The sequences for primer pairs of 16SrRNA Multiplex (mPCR), Semi-nested *Omp1 *(snPCR) and 16SrRNA nested PCR (nPCR) were taken from published literature [19-21]. In mPCR assay, the 16SrRNA coding genes of three Chlamydial species viz:*C. trachomatis, C. pneumoniae *and *C. psittaci *were selected. The primers (Microsynth, Switzerland) were synthesized and were tested for purity by HPLC. The sequences of primers are listed in Table 1.

**Table 1:** Details of oligonucleotide sequences used for detection of *C. pneumoniae *by NAATs.

	**Assays** **Target**	**Format** **Assay**	**Primers**	**Amplicon size (bp)**	**cycle**
1.	16SrRNA	Multiplex			
		*C.pneumoniae*			
			5' TGA CAA CTG TAG AAA TAC AGC 3'	463 bp	30
			5' TAA ATA TCC TCT CTC CGC 3'		
		*C.psittaci*			
			5' TGA CCG CGG CAG AAA TGT CGT 3'		
			5' GAC GTT TCC TCT CTC CGC 3'		
		*C.trachomatis*			
			5' TGA CCG CGG CAG AAA TGT CGT 3'		
			5'GGC GTT CCC TCT CTC CGC 3'		
2.	*Omp1*	Semi-nested			
		(1^st ^pair)			
			5' TGC CAA CAG ACG CTG GCG T 3'		35
			5' TCT GAA CTG ACC AGA TAC GT 3'		
		(2^nd ^pair)			
			5' TGC GAC CAT CAA TTC TCA TG 3'	360 bp	30
			5' TCT GAA CTG ACC AGA TAC GT 3'		
3.	16SrRNA	Nested			
		(1^st ^pair)			
			5' GCT GGC GGC GTG GAT G 3'		30
			5' CGA CAC GGA TGG GGT TG 3'		
		(2^nd ^pair)			
			5' TGG CGG AAG GGT TAG TAG TA 3'	570 bp	30
			5' CCC TTT TCC CCA TCT ATC C 3'		

### Nucleic Acid Amplification Tests (NAATs)

All PCR reactions were performed on a Peltier based thermocycler (Eppendorf, Germany), amplicon were electrophoresed on agarose gels stained with ethidium bromide (0.5 pg/ml) and visualized on Gel documentation system (Alpha Imager, San Laendro, USA). Positive and negative controls were included in all PCR reactions.

#### i) Multiplex PCR (mPCR)

mPCR was used for the detection of *C. pneumoniae*, *C. trachomatis *and C. *psittaci *in blood of CAD patients and controls. The PCR final reaction volume of 50 μl contained 0.2 μM primers, 0.2 mM deoxynucleotide triphosphates, lX PCR buffer (10 mM Tris [pH 8.3], 50 mM KCI, 2.5 mM MgCl_2_, 0.01% gelatin), and 1 U of *Taq *polymerase (Invitogen, USA). Samples were subjected to 30 cycles of denaturation (94°C, 1 min), annealing (55°C, 1 min), and extension (72°C, 1 min). Amplified product was electrophoresed on 1.2% agarose.

#### ii) Nested PCR (nPCR)

For performing nested PCR, amplicon contamination prevention measures were strictly applied as described by Kwok and Higushi [22].

##### a) *Omp1 *snPCR

*Omp1 *snPCR antisense primer was same for both outer and inner sets of reactions. In the first step PCR was performed in 50 μl reaction containing 0.25 uM primers, 0.2 mM deoxynucleotide triphosphates, lX PCR buffer (10 mM Tris [pH 8.3], 50 mM KCI, 2.5 mM MgCl_2_, 0.01% gelatin), and 1 U of *Taq *polymerase (Invitogen, USA). Samples were subjected to 35 cycles of denaturation (94°C, 1 min), annealing (55°C, 1 min), and extension (72°C, 1 min) for amplification of outer pairs. In the second step, 5 μl of sample from step one was amplified using same conditions in 30 cycles

### nPCR

The first step of nPCR was performed in a 50 μl reaction mixture, containing 0.20 uM primers, 0.2 mM deoxynucleotide triphosphates, lX PCR buffer (10 mM Tris [pH 8.3], 50 mM KCI, 3.0 mM MgCl_2_, 0.01% gelatin), and 1 U of *Taq *polymerase (Invitogen, USA). Cycling parameters were of 30 cycles of denaturation at 94°C for 30 s, annealing at 60°C for 60 s and elongation at 72°C for 90 s. In the second step, 5 ul of first step PCR product of 570 bp was amplified in 30 cycles of denaturation at 94°C for 30 s, annealing at 68°C for 60 s and elongation at 72°C for 60 s. We further evaluated the efficiency of nPCR to detect *C. pnuemoniae *in lowest amount of patient DNA. Various concentrations of DNA viz. 25, 50, 75 and 100 ng were taken and amplification was carried out as described above.

### Serology

The detection of *C. pneumoniae *specific IgG and IgA was performed using an ELISA kit (R-Biopharm AG, Germany) as per manufacturer's instructions. This is done on the basis of index number, which is obtained by dividing the absorbance for the sample by the calculated average cut off control value. An index value >1.1 is predicted as *C. pneumoniae *positive; 0.9–1.1 as equivocal; <0.9 as *C. pneumoniae *negative. All calculations have been done considering only true positive *C. pneumoniae *cases.

### Statistical analysis

SPSS version 12.0 for Windows (SPSS Inc., Chicago, USA) was used for statistical testing. All PCR results were dichotomized as positive or negative. For comparing diagnostic assays, the Fisher-exact statistic for binary related variables was used. For the comparisons of results, pairs of assays, the two highest-ranking diagnostic assays were compared with all others by using McNamara's test of two related variables. Simultaneously, an alpha level of 0.05 was set as the level of significance. To evaluate baseline clinical characteristics binary logistic regression was used.

## Results

The mean age for CAD patients and healthy controls were comparable (54.19 and 51.34 yrs respectively). Out of 91 CAD patients, 72 were males and 19 were females, while in 46 healthy controls 36 were males and 10 females.

**Figure 1:**
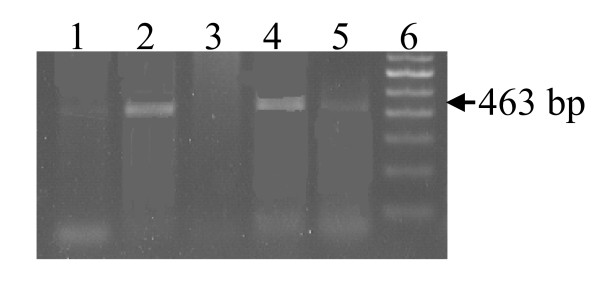
mPCR for 16SrRNA *C. pneumoniae *specific gene. Amplified product was electrophoresed on 1.2% agarose gel yielding a 463 bp product. Lane 1 Positive control, Lane 2, 4, 5 DNA of CAD patients, Lane 3 negative control and Lane 6, 100 bp ladder.

**Figure 2:**
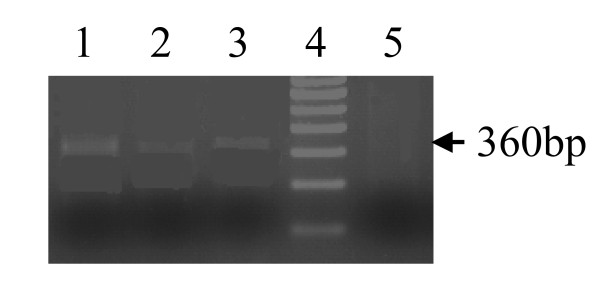
snPCR for *Omp1 C. pneumoniae *specific gene. Amplified product was electrophoresed on 1.5% agarose gel yielding a 360 bp product. Lane1 positive control, Lane 2&3 DNA of CAD patients, Lane 4, 100 bp ladder, Lane 5, negative control.

**Figure 3:**
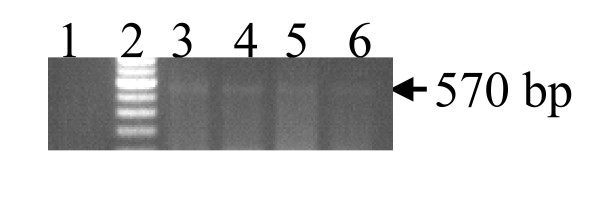
nPCR for 16SrRNA gene specific for *C. pneumoniae*. Amplified product was electrophoresed on 1.2 % agarose gel yielding a 570 bp product. Lane 1 Negative control, Lane 2 100 bp ladder Lane 3 positive control, Lane 4, 5, 6, CAD patients DNA

**Figure 4:**
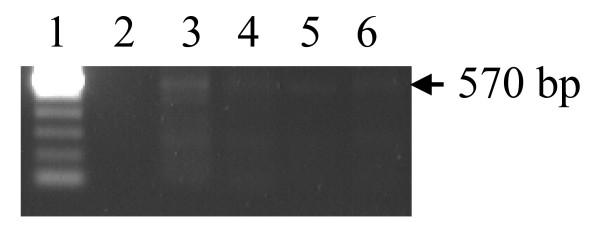
Quantitative nPCR for 16SrRNA gene specific for C. pneumoniae. Amplified product was electrophoresed on 1.2 % agarose gel yielding a 570 bp product. Lane 1 100 bp ladder, Lane 2 negative control, Lane 3,4,5 and 6, CAD patients DNA 100, 75, 50, 25 ng respectively.

Results of agarose gel electrophoresis on amplified products for mPCR, snPCR and nPCR are shown in Figures 1, 2, and 3. In mPCR, positivity for *C. pneumoniae *was higher in CAD patients (6.5%) compared to 0% in healthy controls (p = 0.08). The specificity of mPCR for *C. pneumoniae *was confirmed using positive control DNA of *C. trachomatis and C. psittaci*. In snPCR and nPCR positivity for *C. pneumoniae *was higher in CAD patients 13.8% and 29.67% respectively in comparison to healthy controls with percentage positivity 4.3%, 10.86% respectively (p = 0.125; p = 0.031) (Table 2). The sensitivity and specificity of snPCR as compared to nPCR were 37.03%, 96.96% and the positive predictive value (PPV) and negative predictive value (NPV) were 79.01 and 29.03 respectively, which were significant (p = 0.001) (Table 3). When mPCR was compared with nPCR the sensitivity and specificity were 22.22%, 100% respectively, and the PPV and NPV were 100, 76.19 respectively, (p > 0.001) (Table 3). In quantitative nPCR as low as 25 ng of patient DNA showed amplification at 30 cycles (Figure 4).

**Table 2:** Prevalence of *C. pneumoniae *in CAD patients using NAATs.

NAATs	CAD Pt. C.p +ve (N = 91)	Healthy Control C.p +ve (N = 46)	P Value
nPCR	27 (29.67)	5 (10.86)	0.031*
Omp1snPCR	12 (13.18)	2 (4.3)	0.125
mPCR	6 (6.5)	0 (0)	0.08

Figure in parenthesis indicates % positivity,

C.p = *C. pneumoniae*,

* Statistical significance of % positivity of *C. pneumoniae *in CAD patients compared to healthy controls.

**Table 3:** Comparison of nPCR (gold standard) with snPCR and mPCR.

NAAT	Sensitivity (%)	Specificity (%)	PPV	NPV	Prevalence (%)	P Value
snPCR Vs nPCR	37.03	96.96	83.33	79.01	29.03	0.001*
mPCR Vs nPCR	22.22	100	100	76.19	29.67	>0.001*

* The above table shows the performance of nested PCR with respect to semi nested and multiplexes PCR. The level of significance (P Value) has been calculated using McNamara's test.

Taking nPCR as gold standard the baseline clinical characteristics age, sex, smoking habit, alcohol consumption and family history of CAD, blood pressure and diabetes were compared in *C. pneumoniae *positive CAD patients and controls. No significant variation in % positivity for *C. pneumoniae *was observed in male v/s female, however, a significant difference in % positivity for *C. pneumoniae *in heavy smokers CAD patients (p = 0.019) was observed when compared to healthy controls with odds ratio of 4.57, 2.63 and 1.19 in heavy smokers, occasional smokers and never smoker respectively (Table 4). Percent Positivity for *C. pneumoniae *among non-alcoholic CAD patients was significantly higher (p = 0.026) compared with the other groups, the odds ratio for heavy, occasional, never alcoholic was 1.24, 1.60 and 4.75 respectively. No significant difference in % positivity of *C. pneumoniae *was detected in patients with family history of heart disease, high BP or diabetes; the odds ratio was 2.64, 2.91 and 3.00 respectively.

**Table 4:** Comparison of baseline clinical characteristics of *C. pneumoniae *positive CAD patients and healthy controls.

	CAD pt	Healthy Control	O.R.	
	C.p +ve	C.p +ve		P value
(Mean) Age, yr.	54.19	51.34		NS
Sex				
Male (%)	23(31.9)	04(11.1)	2.87	NS
Female (%)	04(21.1)	01(10.0)	2.10	NS
Smoker				
Heavy (%)	17(65.3)	02(14.2)	4.57	0.019*
Occasional (%)	05(26.3)	01(10.0)	2.63	NS
Never (%)	05(10.8)	02(09.0)	1.19	NS
Alcohol				
Heavy (%)	05(16.6)	02(13.3)	1.25	NS
Occasional (%)	03(20.0)	01(12.5)	1.60	NS
Never (%)	19(41.3)	02(08.6)	4.75	0.026*
Family History	05(29.4)	01(11.1)	2.64	NS
Of CAD (%)				
History of BP (%)	05(41.6)	01(14.2)	2.91	NS
History of Diabetes (%)	05(50.0)	01(16.1)	3.00	NS

All groups are mentioned in absolute numbers and corresponding percentages in parentheses. Total number of subjects for both the group is different in various groups, C.p = *Chlamydia pneumoniae*, +ve = positive, O.R. = odds ratio.

**Figure 5:**
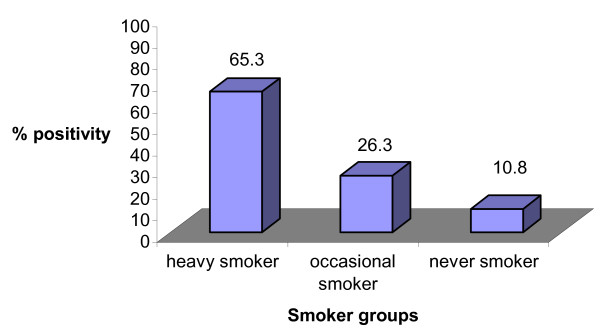
Graph showing % positivity of *C. pneumoniae *in CAD patients with different smoking habits. On Y axis % positivity for *C. pneumoniae *positivity is depicted. On X-axis; smoker groups are divided into heavy smoker, occasional smoker and never smoker.

**Figure 6:**
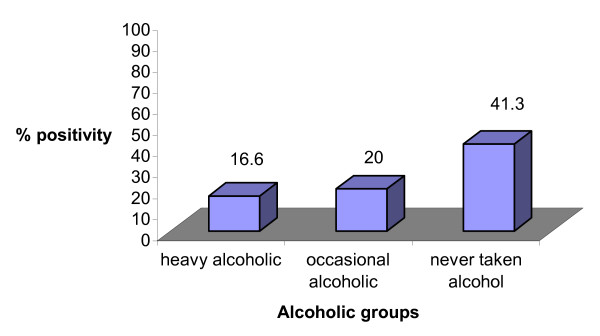
Graph showing % positivity of *C. pneumoniae *in CAD patients with different alcohol consumption. On Y axis % positivity for *C. pneumoniae *is depicted. On X-axis- alcoholic groups divided into heavy alcoholic, occasional alcoholic and never taken alcohol.

Further, two variables, mainly smoking habit, alcohol consumption was also compared in *C. pneumoniae *positive CAD patients (Figure 5, 6). In CAD patients, % positivity for *C. pneumoniae *was significantly higher in heavy smokers group as compared to occasional or never smoker (p = 0.017, 0.003) (Figure 5). In non-alcoholic CAD patients, % positivity for *C. pneumoniae *was significantly higher (p = 0.001) (Figure 6) compared to occasional (p = NS) or heavy alcoholic.

**Figure 7:**
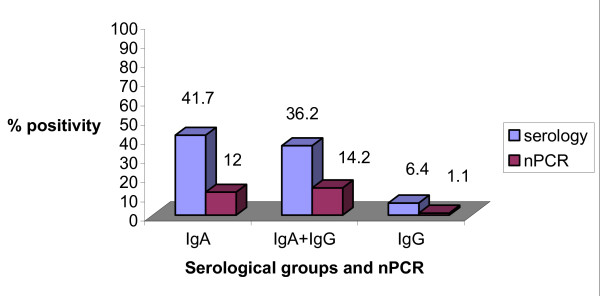
Graph showing percentage of *C. pneumoniae *positivity in CAD patients with serology group IgA, IgA+IgG, IgG and with nPCR. On Y axis % positivity in CAD patient for *C. pneumoniae *is depicted. On X-axis serological marker IgA, IgA+IgG, IgG with nPCR.

In (Figure 7) nPCR positivity in blood of CAD patients were compared with positivity for *C. pneumoniae *specific IgA, IgA+IgG and IgG antibodies. Out of 29.67% nPCR *C. pneumoniae *positive CAD patients, 12% were positive for *C. pneumoniae *specific IgA, 14.2% for both IgA+IgG and 1.1% for IgG. In controls, out of 10.8% nPCR positive individuals, 4.3% were positive for *C. pneumoniae *specific IgA and also for IgA+IgG antibodies. None of the nPCR positive controls had *C. pnuemoniae *specific IgG antibodies.

## Discussion

Chronicity of persistent infection of *C. pneumoniae *may be a major factor associated with the pathophysiological changes leading to CAD. In India there is an urgent need for sensitive methods that could detect and provide direct evidence of *C. pneumoniae *in CAD patients and identify chronic/persistent infections. Serology may be more predictive in detecting a previous or an ongoing *C. pneumoniae *infection, while PCR is an alternative for a rapid identification of this pathogen in circulating blood [23,24].

Although NAATs offer the promise of exquisite sensitivity, theoretically allowing for detection of a single organism in a clinical sample, both false-negative and positive results can and do occur. The reported positivity rates for detection of *C. pneumoniae *DNA by various NAATs ranged from 0% to 60% [25]. Reports suggest that there is poor concordance between different laboratories, as only 25% agree on one specimen and there is no correlation between the detection rates and the sensitivity of NAATs used.

Keeping these observations in mind all good laboratory practice guidelines were followed according to Kwok and Higushi [22]. Among NAATs used in this study, nPCR showed higher sensitivity and specificity than mPCR or snPCR with its percentage positivity of 29.67%. Our results also indicate that nPCR is highly sensitive to detect as low as 25 ng of DNA from CAD patients. In control group (consisted of hospital-based workers) 10.8 % positivity for *C. pneumoniae *was detected by nPCR and this could have reflected an overestimation of the occurrence of *C. pneumoniae *in a healthy population since hospital-based workers are more likely to be exposed to individuals with respiratory tract infections.

Previously it has been reported that increased IgA antibody levels reflects an active or persistent infection, whereas IgG levels reflect only a previous or past infection with *C pneumoniae *[26]. Our data also suggest that those CAD patients having IgA antibodies in their serum indicate a persistent or active infection while patients having both IgA and IgG may reveal chronicity of persistent active infection and the presence of IgG alone reflects past infection. It has been reported that serum IgA antibody to *C. pneumoniae *may be more predictive of coronary heart disease than IgG because IgA antibodies have different biological characteristics and may be better indicators of persistent chlamydial infection [27,28]. The indication for the past infection of C.*pneumoniae *was detected in four individuals in whom both nPCR and IgG were positive but were negative for IgA. It is interesting to note that in two CAD patients *C. pneumoniae *was detected by nPCR, however *C. pneumoniae *specific IgA and IgG antibodies were not detected. This could indicate an acute infection where antibodies responses are yet to occur [29]. In our study out of 71 IgA positive CAD patients, 51 were negative by nPCR. It is reported that infection with *C. pneumoniae *is common, since seroepidemiological studies demonstrated that 50 to 70% of adults have antibody to *C. pneumoniae *and that nearly everyone acquires at least one *C. pneumoniae *infection during his or her lifetime and always it may not lead to symptomatic disease [24].

Earlier reports suggested that males are more prone to CAD as well as *C. pneumoniae *infection [30], which has also been demonstrated in our study. Further *C. pneumoniae *was detected significantly more in CAD patients who were heavy smokers followed by occasional smokers or non-smokers showing thereby that heavy smokers are more prone to *C. pneumoniae *infection. Most of the proposed proatherogenic actions of smoking, such as interference with blood coagulation, induction of endothelial dysfunction, and promotion of lipid peroxidation, reverse themselves shortly after cessation of smoking. Such a pathomechanism may be relevant to the development of vessel pathology among smokers because of the facilitating effects of smoking on the manifestation of various types of persistent infectious illness [12,31]. CAD patients who were non-alcoholic showed significant higher positivity for *C. pneumoniae *than occasional or heavy alcoholic. The mechanism by which alcohol consumption reduces the risks for CAD appears to be multifactorial and remains conjectural [32]. Although it has been reported that long-term ethanol feeding reduces the severity of early atherosclerotic lesions in mice and ethanol inhibits human platelet activation and enhances endothelial fibrinolytic activity both of which are important for athrombogenesis [33]. Further, we detected the higher prevalence of *C. pneumoniae *in CAD patients who have had family history of diabetes and high blood pressure than controls showing thereby they are at higher risk of having CAD.

## Conclusion

In conclusion, high prevalence of *C. pneumoniae *in CAD patients as revealed by nPCR in our country is a matter of concern. Positive nPCR findings in conjunction with high *C. pneumoniae *specific antibody prevalence may suggest an ongoing infection. Further studies are needed to define and determine the clinical and prognostic implications of such findings.

## List of abbreviations

Ig = Immunoglobulins, ELISA = Enzyme Linked Immunosorbant Assay, PCR = Polymerase Chain Reaction, NAATs = Nucleic Acid Amplification Tests, Omp1= Outer Membrane Protein, CAD = Coronary Artery Disease, NS = Non Significant, BP = Blood Pressure, C.p = *C. pneumoniae*, +ve = positive, O.R. = Odds Ratio.

## Competing interests

The author(s) declare that they have no competing interests.

## Authors' contributions

Jha HC, Harsh, Gupta R and Mittal A contributed to data interpretation and writing of the paper. Jha HC, Prasad J and Varma R contributed to analysis of baseline clinical characteristic CAD patients with respect to controls.

## Pre-publication history

The pre-publication history for this paper can be accessed here:


